# Signal amplification method for miR-205 assay through combining graphene oxide with duplex-specific nuclease

**DOI:** 10.1039/c9ra04663a

**Published:** 2019-08-30

**Authors:** Zhaoqi Yang, Lan Qin, Dutao Yang, Weixia Chen, Yue Qian, Jian Jin

**Affiliations:** School of Pharmaceutical Sciences, Jiangnan University Wuxi 214122 China zhaoqiyang@jiangnan.edu.cn jinjian31@126.com

## Abstract

Since microRNA-205 (miR-205) is a predictive biomarker for anti-radiation of nasopharyngeal carcinoma (NPC), quantitative detection of miR-205 is important for developing personalized strategies for the treatment of NPC. In this investigation, based on the graphene oxide sensor and duplex specific nuclease (DSN) for fluorescence signal amplification, a highly sensitive detection method for miR-205 was designed. A target-recycling mechanism is employed, where a single miR-205 target triggers the cleavage of many DNA signal probes. The method shows the ability to analyze miR-205 in solution, and it can detect miR-205 at concentrations as low as 132 pmol L^−1^ with a linear range of 5–40 nmol L^−1^. Furthermore, the method is specific in that it distinguishes between a target miRNA and a sequence with single base, double base and three base mismatches, as well as other miRNAs. Considering simplicity and excellent sensitivity/specificity, it is promising for applications in radioresistance studies as well as the early clinical diagnosis of NPC.

## Abstract 2

A signal amplified method for detecting a biomarker of radiation-resistant nasopharyngeal carcinoma using graphene oxide and duplex-specific nuclease was constructed.

## Graphical abstract

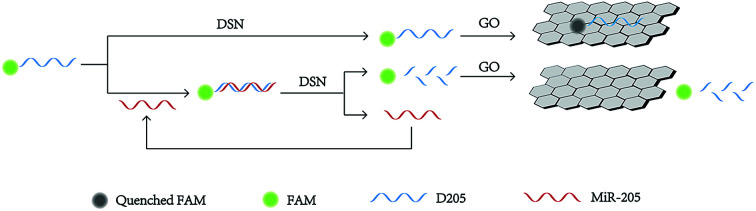

## Introduction

Nasopharyngeal carcinoma (NPC) is an endemic disease prevalent in Southeast Asia.^1^ Radiation therapy has become the standard treatment for locally advanced NPC due to the radiosensitivity of the top and side walls of the nasopharyngeal cavity.^2,3^ However, the failure of radiotherapy for NPC will lead to treatment delays.^4,5^ It can be seen that the problem of radiation resistance needs to be solved urgently.

MicroRNAs (miRNAs) are a class of endogenous non-coding single-stranded RNA found in eukaryotic cells, usually 19 to 25 nucleotides in length, about 30% of human genes are regulated by miRNAs, and the expression levels of miRNAs are closely related to the occurrence of various diseases in humans.^6,7^ Studies have shown that the abnormal expression of miRNAs is closely related to the emergence of a variety of cancers, and can be used as an effective biomarker for the diagnosis, progression and therapeutic response of cancer.^8–12^ miR-205, also known as hsa-miR-205, is micro-RNA which includes 22 bases. In the gene sequence based on the protection of *Takifugu rubripes* and mice, miR-205 was first predicted by computer and subsequently confirmed in zebra fish and humans. Recent studies have shown that miR-205 is significantly higher in NPC radiation-resistant cells than normal NPC cells,^13,14^ indicating that miR-205 is associated with radioactivity in NPC cells. miR-205 may be used as a biomarker to predict radioresistance in NPC. This finding may contribute to the personalized treatment of NPC, thus the method of detecting miR-205 with high sensitivity is of great significance.

Currently, there are many widely used methods developed for the detection of miRNAs, including northern blotting,^15^ quantitative real-time polymerase chain reaction (qRT-PCR),^16^ microarray analysis^17^ and so on. The northern blot method has the disadvantages of large sample volume, cumbersome operation, long time consumption, multiple interference factors, and radioactive contamination. Due to the short length of miRNAs, the design of amplification primers is very difficult, and most of the traditional PCR techniques are indirectly judged by examining their precursors. Therefore, qRT-PCR does not reflect the true level of miRNAs. Microarray technology enables simultaneous detection of multi-component miRNAs. However, the fabrication and detection steps of the chip are cumbersome and costly, and cross-hybridization is prone to occur.^18^

Graphene oxide (GO) is a two-dimensional nanomaterial. Due to its good dispersibility and hydrophilicity, high cell absorption rate, rapid fluorescence quenching ability and abundant modifiable sites, it has been widely used in biomedical fields such as bio-propagation and biosensing.^19–21^ GO is a two-dimensional monolayer carbon material that strongly adsorbs single-stranded oligonucleotide molecules by π–π stacking effect and efficiently quenches the fluorescent signal of fluorescent dyes or fluorescent nanoparticles labeled on oligonucleotides.^22^ When the target miRNA hybridizes with the dye-labeled DNA probe, a heteroduplex is formed that detaches from the GO surface and restores the fluorescent signal.^23,24^ According to the previous studies,^25^ Mn^2+^ will be formation with the generation of GO.

Duplex-specific nuclease (DSN), a nuclease isolated from the hepatopancreas of the crab, Kamchatka, which selectively recognizes and cleaves a perfectly matched DNA duplex or the DNA in the RNA/DNA hybrid double strand has no effect on single-stranded DNA, RNA or double-stranded RNA, and can distinguish between target RNA and single-base mismatched RNA.^26–28^ Therefore, DSN paved a promising way for ultrasensitive and accurate quantitation of miR-205.

Herein, a highly sensitive detection method which in the help of signal amplification has been designed by combining the fluorescence quenching effect of a fluorescently labeled single-stranded DNA probe with GO, and DSN. Compared with common graphene oxide sensors, this method has higher sensitivity and higher selectivity, and it has broad application prospects in the analysis of biomarkers for predicting radiation tolerance in NPC.

## Experimental section

### Materials and reagents

Sodium chloride (NaCl), magnesium chloride hexahydrate (MgCl_2_·6H_2_O) and Tris were purchased from China National Pharmaceutical Group Corp. GO was purchased from Nanjing XFNANO Materials Tech. Co., Ltd. (Nanjing, China). A DSN kit containing 100 U DSN and 10× DSN reaction buffer was purchased from Evrogen (Moscow, Russia). Fluorescent aptamer (D205) and target RNA were synthesized by Sangon Biotechnology Co. Ltd. (Shanghai, China) and purified through high-performance liquid chromatography (all sequences used in this investigation show in Table 1). The 1× Tris–HCl buffer solution (20 mmol L^−1^ Tris–HCl, 100 mmol L^−1^ NaCl, 10 mmol L^−1^ MgCl_2_·6H_2_O, pH = 7.2) was prepared using DEPC water. Fluorescence spectra were obtained from Hitachi F-7000 spectrophotometer (Hitachi Ltd., Japan). The samples placed in quartz cuvettes were excited at a wavelength of 480 nm and all emission spectra were collected at wavelengths ranging from 500 to 600 nm at room temperature.

**Table 1:** Nucleic acid sequences use in this investigation

Name	Sequence (5′–3′)
D205	FAM-CAGACTCCGGTGGAATGAAGGA
miR-205	UCCUUCAUUCCACCGGAGUCUG
Rm1 (single-base mismatch)	UCAUUCAUUCCACCGGAGUCUG
Rm2 (double-base mismatch)	UCAUUCAUUCAACCGGAGUCUG
Rm3 (three-base mismatch)	UCAUUCAUUCAACCGGAGUAUG
miR-21	UAGCUUAUCAGACUGAUGUUGA
miR-141	UAACACUGUCUGGUAAAGAUGG
Let-7a	UGAGGUAGUAGGUUGUAUAGUU
NC (non-complementary RNA)	UUGUACUACACAAAAGUACUGA

### Maximization of enzyme action

In order to promote the optimal function of the DSN enzyme, the various conditions which including the order of GO addition, the amount of DSN, the temperature of the reaction solution and the time required for the reaction were separately optimized.

For the exploration of the operation steps, two methods of operation were tried respectively. Method one, D205 (100 nmol L^−1^) was added to 10 μg mL^−1^ of GO solution for 10 minutes, then miR-205 (50 nmol L^−1^) and DSN (0.3 and 0.6 U) was added into react mixture at 52 °C for 3.5 hours. Method two, DSN (0.3 and 0.6 U) was added to buffer containing D205 (100 nmol L^−1^) and miR-205 (50 nmol L^−1^) at 52 °C for 3.5 hours, and finally GO (10 μg mL^−1^) was added for 10 minutes.

Regarding the optimization of the amount of DSN, DSN (0.1, 0.2, 0.3, 0.4, 0.5, 0.6 and 0.7 U) was added to buffer containing D205 (100 nmol L^−1^) and miR-205 (50 nmol L^−1^) at 52 °C for 3.5 hours, and finally GO (10 μg mL^−1^) was added for 10 minutes.

As for the determination of the detection temperature, DSN (0.3 U) was added to buffer containing D205 (100 nmol L^−1^) and miR-205 (50 nmol L^−1^) at different temperature (49, 52, 55 and 58 °C) for 3.5 hours, and finally GO (10 μg mL^−1^) was added for 10 minutes.

The reaction time is equally important, thusDSN (0.3 U) was added to buffer containing D205 (100 nmol L^−1^) and miR-205 (50 nmol L^−1^) at 52 °C for different times (0, 0.5, 1, 1.5, 2, 2.5, 3, 3.5 and 4 hours), and finally GO (10 μg mL^−1^) was added for 10 minutes.

### miRNA-205 detection

Various concentrations of miRNA-205 (0–150 nmol L^−1^) were added to 100 nmol L^−1^ D205, followed by 0.3 U DSN and a quantity of 10× DSN buffer were added. After incubation at 52 °C for 3.5 hours, 10 μg mL^−1^ of GO was added for 10 minutes. The fluorescence intensity was excited at 480 nm and measured at 520 nm.

### Specificity experiments

Three mismatches and two different target sequences were designed, which including single base mismatch targets (Rm1), double base mismatch targets (Rm2), three base mismatch targets (Rm3), NC (non-complementary RNA), miR-21, miR-141 and Let-7a. The RNA sequence and D205 were mixed while reacting with DSN for 3.5 hours, then GO was added for 10 minutes, and the fluorescence intensity at 520 nm was recorded, and the *F*/*F*_0_ ratio was compared.^29–32^

## Results and discussion

### Detection principle

**Scheme 1:**
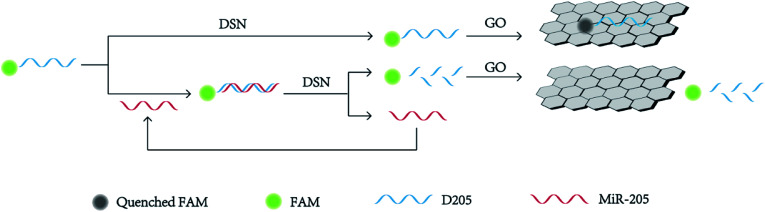
Schematic representation of miR-205 detection based on DSN-assisted signal amplification.

Scheme 1 shows the strategy of detecting miR-205 under assistant of signal amplified by DSN. GO could bind single-dye-labeled ssDNA probe by the π–π stacking effect between the ring structure of nucleobases and the hexagonal cells of the GO. The sp^2^ aromatic domains within GO induced efficient fluorescence quenching of the dyes *via* fluorescence resonance energy transfer (FRET). In the absent of miR-205, ssDNA is adsorbed on the GO surface and the fluorescence is quenched. On the other hand, in the presence of miR-205, DNA/RNA heteroduplexes were formed as the substrate for DSN. DSN selectively hydrolyzed DNA strands in DNA/RNA heteroduplexes, resulting in the release of miR-205 and the recovery of fluorescence signal. Because miRNA-205 remained intact during the hydrolytic process, it could return to the solution and then react with another DNA, which trigger the next round of DSN-based hydrolytic process. Such a cycle started anew, leading to the continuous cleavage of DNA/RNA heteroduplexes, which greatly accumulated the fluorescence signal for miRNA-205 detection.^33–35^ As a consequence, the change of fluorescence intensity with the concentration of miR-205 could be measured correspondingly. It was speculated by us that the purposed method was suitable for using to determination of miR-205.

### Feasibility analysis

**Fig. 1:**
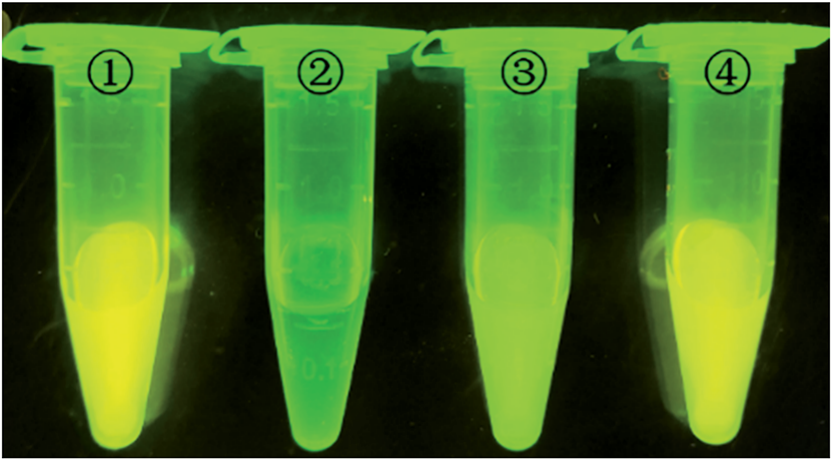
Photograph of vials containing (1) D205, (2) D205/GO, (3) D205/GO/miR205 and (4) D205/GO/miR205/DSN expose in UV light. The concentration of D205 in each sample was 100 nmol L^−1^, the concentration of GO was 10 μg mL^−1^, the concentration of miR-205 was 50 nmol L^−1^, and the amount of DSN was 0.3 U, which was reacted at 52 °C and pH 7.2.

In order to initially evaluate the feasibility of the strategy (Fig. 1), four react conditions were performed and results can be definitely observed by the naked eye. Tube 1 is 100 nmol L^−1^ D205, tube 2 is mixed with 100 nmol L^−1^ D205 in 10 μg mL^−1^ GO solution, tube 3 add 50 nmol L^−1^ target miR205 to GO (10 μg mL^−1^) containing 100 nmol L^−1^ D205, tube 4 was further added with 0.3 U of DSN enzyme in a GO (10 μg mL^−1^) solution containing 100 nmol L^−1^ of D205 and 50 nmol L^−1^ of miR-205. It is easy to find that the fluorescence intensity of tube 2 is almost total quenched by GO. Furthermore, the fluorescence intensity varies greatly in the absence and the presence of DSN. In the absence of DSN (tube 3), the fluorescence intensity is slightly restored, which owing to the fluorescence only contributed by the concentration of D205. On the other hand, the fluorescence intensity obvious recovers to the initial state under the action of DSN (tube 4). Because DSN specifically digest DNA in DNA/RNA duplexes which induce RNA cyclic hybridize to complementary DNA probe, fluorescence signal would quickly accumulate and remarkably enhance. These results indicated that the DSN-based GO sensor can achieve signal amplification for detection applications.

### Optimization of the assay conditions

**Fig. 2:**
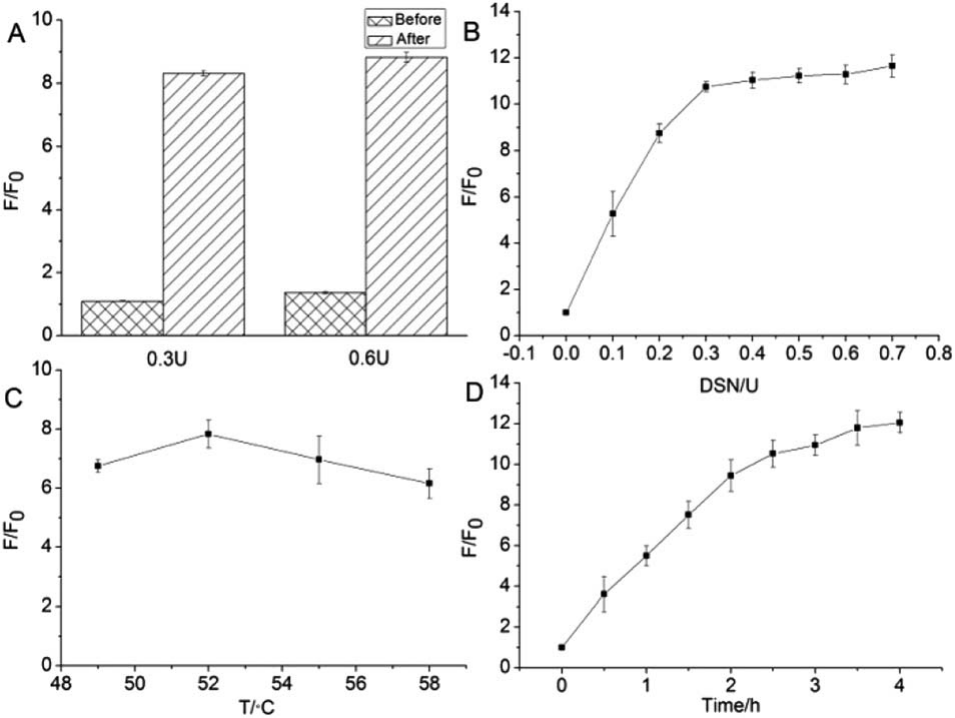
Optimization of the experiments for various DSN react conditions. (A) Effect of different sequence of add GO to DSN digestive reaction, (B) amount of DSN, (C) DSN reaction temperature, (D) DSN reaction time. *F* is the fluorescence intensity under the respective target, and *F*_0_ corresponds to the fluorescence intensity of the system in the absence of the target (*n* = 3).

To optimize the degree of amplification of the sensor signal, various DSN reaction conditions were tested. Considering that GO possesses significantly different adsorption affinity for ssDNA and DNA/RNA heteroduplexes, the effect of different sequence of add GO to the reaction is preferentially investigated. As shown in Fig. 2A, it is clear that the degree of signal amplification is significantly higher in the case which GO was added in the last step. In addition, regardless of the amount of DSN was 0.3 U or 0.6 U, same signal amplified tendency were observed. Consequently, the strategy that GO was put into reaction mixture in the last step was used in the following RNA detection studies.

The enzymatic amplification efficiency is closely related to the amount of DSN. Therefore, the detection sensitivity of miR-205 is inevitably affected by the enzyme content in the system. The effect of different concentrations of DSN (0.1, 0.2, 0.3, 0.4, 0.5, 0.6, 0.7 U) system on the fluorescence response signal was investigated in this experiment. As can be seen from Fig. 2B, as the amount of enzyme increases, the *F*/*F*_0_ ratio increases rapidly, and then maintains a relatively stable trend. When the amount of DSN is more than 0.3 U, further increase in the amount of the enzyme does not cause a markedly signal change. It is demonstrated that 0.3 U DSN is enough to amplify fluorescence signal in the designed detecting platform. As a consequence, 0.3 U was selected as the optimum DSN amount in this experiment.

With the purpose to increase the efficiency of detection, the temperature of digest reaction was also investigated. According to the product specification, the optimum temperature of the DSN is 45–60 °C. Either temperature is too high or too low, the activity of the enzyme will be significantly reduced. Considering the melting temperature (*T*_m_ value) of D205 probe and miR-205 hybrid double-stranded structure, it was optimized at 49–58 °C to ensure efficient hybridization-DSN digestion cycle amplification. As shown in Fig. 2C, when the temperature is 52 °C, the *F*/*F*_0_ ratio reaches the maximum value. However, the signal of the sample system dramatic decline when the temperature over 52 °C. It is speculated by us that D205 and miR205 is difficult to format a stable heteroduplexes resulting from temperature is very close to the *T*_m_ value. The enzymatic cleavage effect observably decreases because there is not enough substrate to perform the digest reaction. Therefore, 52 °C was selected as the detecting temperature in this study. Finally, reaction time was another key factor affecting the enzymatic cleavage reaction and was tested (Fig. 2D). As the reaction time prolonged, the *F*/*F*_0_ ratio gradually increased and revealed that 3.5 hours was the optimal time for the reaction.

### Sensitivity of assay miR-205 by DSN amplified signal

**Fig. 3:**
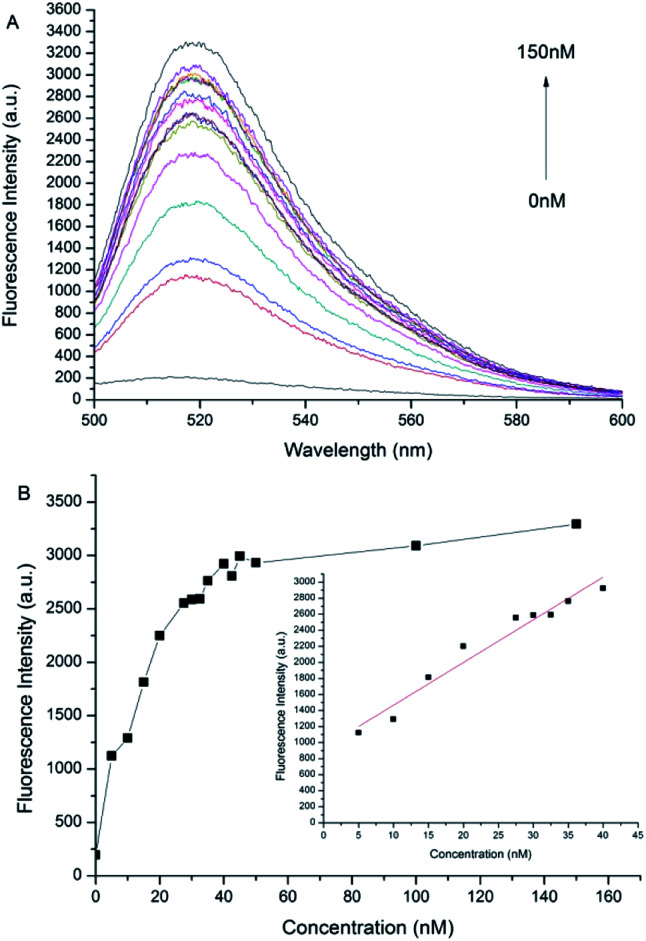
The fluorescence intensities of different concentrations of miR205 were recorded. (A) The concentration of miR205 decreases from top to bottom: 0, 5, 10, 15, 20, 27.5, 30, 32.5, 35, 40, 42.5, 45, 50, 100, 150 nmol L^−1^. (B) The relationship between fluorescence intensity and concentration of miR205.

The results of the proposed method for detecting miR-205 under optimal optimization conditions are shown in Fig. 3. The Fig. 3A is the fluorescence spectrum corresponding to different concentrations of the target miR-205. It is clear to know that almost no fluorescence signal is generated in the absence of miR-205, and the fluorescence intensity of the system gradually enhance when the concentration of miR-205 is increasing. The maximum fluorescence intensity is always exhibited at 520 nm. When the concentration of miR-205 is in the range from 5 to 40 nmol L^−1^, the fluorescence intensity at 520 nm is linear with the concentration (Fig. 3B). The regression equation is *F* = 53.24 × [miR-205] nmol L^−1^ + 933.59, *R*^2^ = 0.954, where *F* is the fluorescence intensity at 520 nm. The detection limit is 132 pmol L^−1^ (3*σ*/slope), where *σ* represents the standard deviation of the control group and the slope represents the slope of the linear regression curve. The detection limit is lower than that of the ordinary GO detection method,^36^ indicating that the combination of DSN and GO sensor can realize fluorescence signal amplification and high sensitivity detection of miR-205.

### Specificity of miR-205 detection by DSN

**Fig. 4:**
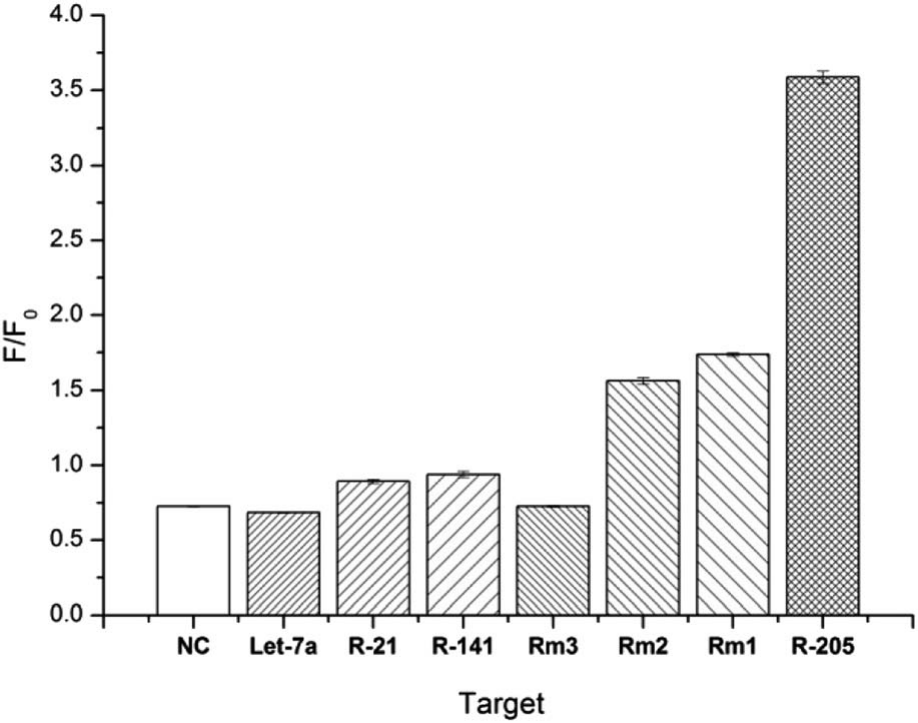
The selectivity of the DSN-GO sensor hybridizes to different miRNA sequences: target miR-205, non-complementary messy RNA (NC), let-7a, miR-21, miR-141, three-base mismatch RNA, two-base error RNA and single base mismatched RNA (*n* = 3).

DSN is a very specific cleavage enzyme that distinguishes base mismatches in double-stranded nucleic acid structures. Based on the superior discriminating power of DSN enzymes, we also examined the specificity of the method. Under the same experimental conditions, when the probe is present, the same concentration of different sequences of miRNAs are detected, including NC, Let-7a, miR-21, miR-141, Rm3, Rm2, Rm1 and miR-205. Interference with RNA detection of miR-205, mismatched target was hybridized to D205 and digested with DSN for 3.5 hours. After 5 minutes of GO was added in reaction, the fluorescence spectrum was recorded and the fluorescence intensity at 520 nm was compared with the fluorescence intensity obtained with miR-205. As shown in Fig. 4, *F*/*F*_0_ decreases as the number of mismatched bases increases, and RNA with one base mismatch has only half of the fluorescent signal change relative to the target RNA, and RNA with more than three base mismatches does not cause significant fluorescence signal changes, which confirms the superior selectivity of our method.

## Conclusions

In summary, after investigating the stability and specificity of molecular probe targeting miR-205 and optimizing the hybridization reaction conditions, a sensitive detection method for miR-205 using the DSN to trigger fluorescence signal cascade amplification is constructed. Through a series of verification, the established method exhibited excellent selectivity, good accuracy and satisfactory sensitivity (LOD, 132 pmol L^−1^), which is expected to provide valuable information for prediction of radioresistance and early clinical diagnosis of NPC.

## Conflicts of interest

There are no conflicts to declare.

## Supplementary Material
